# Barriers to opioid use disorder treatment: A comparison of self-reported information from social media with barriers found in literature

**DOI:** 10.3389/fpubh.2023.1141093

**Published:** 2023-04-20

**Authors:** Whitney Bremer, Karma Plaisance, Drew Walker, Matthew Bonn, Jennifer S. Love, Jeanmarie Perrone, Abeed Sarker

**Affiliations:** ^1^Department of Biomedical Informatics, School of Medicine, Emory University, Atlanta, GA, United States; ^2^Department of Biomedical Informatics, School of Medicine, College of Engineering and Applied Sciences, Stony Brook University, Stony Brook, NY, United States; ^3^Department of Epidemiology, Rollins School of Public Health, Emory University, Atlanta, GA, United States; ^4^Department of Behavioral, Social and Health Education Sciences, Rollins School of Public Health, Emory University, Atlanta, GA, United States; ^5^Canadian Association of People Who Use Drugs, Dartmouth, NS, Canada; ^6^Department of Emergency Medicine, Icahn School of Medicine at Mount Sinai, New York, NY, United States; ^7^Department of Emergency Medicine, Perelman School of Medicine, University of Pennsylvania, Philadelphia, PA, United States

**Keywords:** substance use, substance use disorder, social media, natural language processing, health informatics

## Abstract

**Introduction:**

Medications such as buprenorphine and methadone are effective for treating opioid use disorder (OUD), but many patients face barriers related to treatment and access. We analyzed two sources of data—social media and published literature—to categorize and quantify such barriers.

**Methods:**

In this mixed methods study, we analyzed social media (Reddit) posts from three OUD-related forums (subreddits): r/suboxone, r/Methadone, and r/naltrexone. We applied natural language processing to identify posts relevant to treatment barriers, categorized them into insurance- and non-insurance-related, and manually subcategorized them into fine-grained topics. For comparison, we used substance use-, OUD- and barrier-related keywords to identify relevant articles from PubMed published between 2006 and 2022. We searched publications for language expressing fear of barriers, and hesitation or disinterest in medication treatment because of barriers, paying particular attention to the affected population groups described.

**Results:**

On social media, the top three insurance-related barriers included having no insurance (22.5%), insurance not covering OUD treatment (24.7%), and general difficulties of using insurance for OUD treatment (38.2%); while the top two non-insurance-related barriers included stigma (47.6%), and financial difficulties (26.2%). For published literature, stigma was the most prominently reported barrier, occurring in 78.9% of the publications reviewed, followed by financial and/or logistical issues to receiving medication treatment (73.7%), gender-specific barriers (36.8%), and fear (31.5%).

**Conclusion:**

The stigma associated with OUD and/or seeking treatment and insurance/cost are the two most common types of barriers reported in the two sources combined. Harm reduction efforts addressing barriers to recovery may benefit from leveraging multiple data sources.

## 1. Introduction

The opioid epidemic in the United States (US) has evolved into an alarming public health crisis. 31.9 million Americans over 12 years of age are estimated to be currently involved in substance use (1) and more than 10 million have non-medically used opioids in 2018 (2). Consequent drug overdoses have claimed the lives of over 92,000 Americans in 2020 alone (3), and provisional estimates suggest more than 100,000 overdose deaths occurred in 2021 (4). The opioid epidemic in the US is evolving constantly, and recent surges in overdoses and related deaths have been driven by fentanyl and its analogs (3), stimulants, and novel psychoactive benzodiazepines (5). In addition to mortality, opioids contribute to significant morbidity through opioid use disorder (OUD), which afflicts approximately 1.6 million people in the US (6).

Evidence-based harm reduction efforts for opioid use disorder include interventions such as needle and syringe exchange programs and safe supply initiatives (7–9). Medications for OUD (MOUDs), such as buprenorphine-naloxone and methadone, can mitigate the effects of OUD as per research evidence (10–14). Additionally, long-term MOUD adherence is directly associated with improved outcomes, such as high retention and low relapse rates, significantly lower mortality rates, and lower rates of other opioid use (11). Despite the proven effectiveness and relatively good availability of MOUDs, individuals seeking treatment often face an array of structural and social barriers (15), including the absence of health insurance, high out-of-pocket costs, and a lack of facilities that accept the insurance plans held by patients. Non-insurance barriers such as transportation limitations and stigma surrounding treatment can also discourage or prevent individuals from seeking treatment. The COVID-19 pandemic has amplified barriers to OUD treatment (16) and has exacerbated the existing health disparities in OUD among certain subpopulations, such as racial-ethnic minorities (2, 17). As the epidemic of overdoses associated with substance use and OUD continues to evolve, the barriers associated with treatment are likely to evolve as well. To address barriers to OUD treatment, it is essential to first identify the distinct types of barriers and estimate their impacts.

Harm reduction literature has increasingly highlighted the existence of treatment barriers and disparities. To develop targeted solutions to barriers, it is important to compare findings reported in the literature with barriers reported by those with lived experiences. Data on those experiencing OUD may be hard to ascertain due to factors such as high refusal rates or underreporting. Individuals who are experiencing houselessness or are incarcerated may be missed as well. Due to the difficulty of connecting with this population, their concerns may not be comprehensively represented in the literature, making complementary sources of information desirable. Social media is a potentially rich source of information, often reported by those with lived experiences. Analysis of data from social media could conceivably offer some support for evidence reported in the literature and can provide insight into rapidly changing trends in communities of people who experience substance use (18). Data from social media also have their own limitations. Only a subset of the population is available on social media and analyses of such data are often limited to those who are technologically adept and speak English. Social media samples, including from Reddit, do not often include individuals experiencing houselessness or incarcerated populations, although they may include subpopulations who are not reachable *via* traditional means (e.g., those without health coverage). Social media analytics have been applied to many related topics in recent years including pharmacovigilance (19–22), toxicovigilance (23–26), syndromic surveillance of COVID-19 (27–29), and long-term impacts of COVID infection (long-COVID) (30, 31). Social media data analysis can be done in near real-time, at minimal cost, and with large cohorts. Studies also suggest that social media subscribers may be more willing to share their experiences candidly, given the possibility to remain anonymous on many platforms (e.g., Reddit). In recent works closely related to ours, Chen et al. examined stigma associated with substance use from discussions on Reddit, reporting that internalized stigma was the most commonly expressed, and temporal and affective factors were prominent (32). Reddit was also described as an important source of substance use-related information during COVID-19, particularly because subscribers often shared their concerns and provided social support to each other (33, 34). Studies on specific substances such as cannabis and fentanyl based on Reddit data have also been reported (35, 36). No study, however, has focused specifically on identifying and characterizing barriers discussed on a social network such as Reddit. Social media data, despite its own limitations, have the potential to complement what is known from medical literature by providing an alternative perspective.

In this study, we attempted to obtain a more comprehensive view of the barriers to MOUDs than is currently available by combining two data sources—social media and medical literature. The goal of this study was to conduct a natural language processing (NLP)-driven analysis of selected Reddit posts by community members about OUD treatment barriers and compare the findings to those reported in published literature on the topic.

## 2. Methods

This study was reviewed by the Institutional Review Board (IRB) at Emory University and was deemed to be exempt from review (category 4; publicly available data). No identifiable information is presented in this paper.

### 2.1. Social media data

To find treatment barriers directly reported by the OUD community, we focused on the social network Reddit. Reddit is home to over 52 million monthly active users and is both a prominent and influential data source (37). *Subreddits* are communities within Reddit dedicated to the discussion of targeted topics (e.g., health conditions and medications) and are therefore rich in information and self-reported experiences. Past research has shown that *subreddits* contain a trove of information regarding substance use, typically posted directly and anonymously by people who non-medically use substances (38).

Using the *Python Reddit Application Programming Interface* (API) *Wrapper* (PRAW) (39), we collected all publicly available posts from three subreddits- *r/methadone, r/suboxone* (the primary subreddit for discussions about buprenorphine), *r/naltrexone* that were retrievable *via* the API. We chose these subreddits based on our familiarity with their contents from prior works on substance use (34, 35, 38, 40). These subreddits typically contain discussions between people who are currently taking, have taken in the past, or are considering taking MOUDs. Among other topics, these subreddits also contain discussions about treatment barriers. While these posts provide insights into the experiences of those with OUD, they also serve as a convenience sample, and do not comprehensively represent all the relevant information on the topic within this platform. Before conducting detailed analyses, we manually inspected a small number of posts to verify that there were indeed posts about treatment barriers.

Following the data collection, we applied NLP to detect potential expressions indicating the presence of treatment barriers and then extracted these posts for manual analysis. We included original posts and comments posted in response to them. To identify keywords and phrases that were characteristic of such discussion, we manually curated a set based on our literature review (described in the next subsection). The list of phrases encompassed barriers associated with insurance coverage (e.g., *insurance*), stigma (e.g., *junkie*, *stigma*, *drug [ab]user*), logistics (e.g., *wait time*, *childcare*, *transport*), and treatment facilities and their locations (e.g., *clinic* and *near*). We initially started with a large set of these key phrases and refined them *via* trial and error. This iterative refinement of terms was necessary since we were not aware of the terms and phrases most commonly used to discuss barriers, and there is no lexicon available for this task. After each NLP-driven search for a finite set of expressions, we manually reviewed samples of the retrieved posts and added additional expressions (e.g., potential keywords missed in our initial set) or removed them (e.g., if the phrases brought in too much irrelevant noise).

Our NLP-driven search involved *fuzzy* matching, as opposed to exact matching. Fuzzy matching allows the matching of words and phrases even if they are not identical to the search query. Instead of generating a binary output to indicate a match or not, fuzzy matching generates a similarity value in the range [0:1], with 1 indicating an exact match. We first preprocessed the texts by lowercasing, and removing punctuations and URLs. We also tokenized the posts by splitting them at word boundaries. To compute the similarity between a search expression and a text span within a post, we used the Levenshtein ratio metric, as implemented in the python-Levenshtein package (41). For each search expression, we passed a sliding window of the same length as the expression (in terms of words) through the text of the post, and computed the Levenshtein ratio between the two text segments. The computation of this metric was at the character level (i.e., the words within a window were represented as a single string). This approach enabled us to identify text segments lexically similar to the query expressions so that misspellings were captured in our searches. Expressions with Levenshtein ratios greater than 0.9 were considered to be matches.

Using the NLP methods described, we initially extracted 522 posts for manual review. During the manual review process, we decided to only analyze posts related to personal experiences and did not predetermine the coding categories nor limit the number of posts to include. We sorted the posts into several categories based on recurring themes that we observed. After the categories were determined, we completed a second review to ensure that each post was categorized appropriately and allowed for posts to fit into multiple categories. This method enabled us to identify and understand common barriers to medication treatment that were self-reported through these Reddit posts. This process involved all the authors of this paper, and instead of coding independently by multiple people and then comparing inter-coder agreements, we determined the final categories *via* discussion. We chose this approach because we were not aware of all the possible and relevant categories *a priori.* While it was possible to predetermine the categories based on our review of the literature, such an approach would bias the social media data analysis and potentially exclude categories unique to this source.

We began the review process by omitting unrelated posts (e.g., about alcohol use disorder) and sorting the rest into appropriate categories. In the first iteration of the review, the posts were sorted into two major categories—non-insurance-related barriers and insurance-related barriers. The non-insurance-related barriers were further sorted into six subcategories and the insurance-related barriers were sorted into five.

### 2.2. Literature review

To find a sample of relevant publications for review, we conducted PubMed searches using the keywords *barriers* + *substance [ab]use medication* + *substance [ab]use treatment*. The results returned by our initial searches were narrowed down to 362 publications based on relevance and years of publication (2006–2022). Conducting a full systematic review of the topic was not within the scope of this study. Instead, the objective was to include a small set of important and representative papers so that we could compare the findings with those obtained from Reddit. We then chose publications that had the word(s) *barrier*, *challenges*, and *needs* (which allude to the presence of barriers) in the title, abstract, or keywords. Each publication was reviewed for mentions of non-insurance-related barriers as well as detailed expressions (e.g., “*Stigma [of being a] mother [and an] addict*,” “*wait time*,” and “*admission/admitted*”). We also attempted to identify the affected populations (i.e., their demographic characteristics) mentioned in the studies to identify OUD subpopulations.

## 3. Results

### 3.1. Social media data

The retrieved posts ranged from January 2014 to August 2021. From the 522 initial posts, 31 unrelated posts about alcohol use disorder were found in the subreddit *r/naltrexone* in addition to 62 and 167 other unrelated posts within the non-insurance-related and insurance-related barrier posts, respectively. After these exclusions, we included in our analysis 178 insurance-related and 84 non-insurance-related posts.

**Figure 1:**
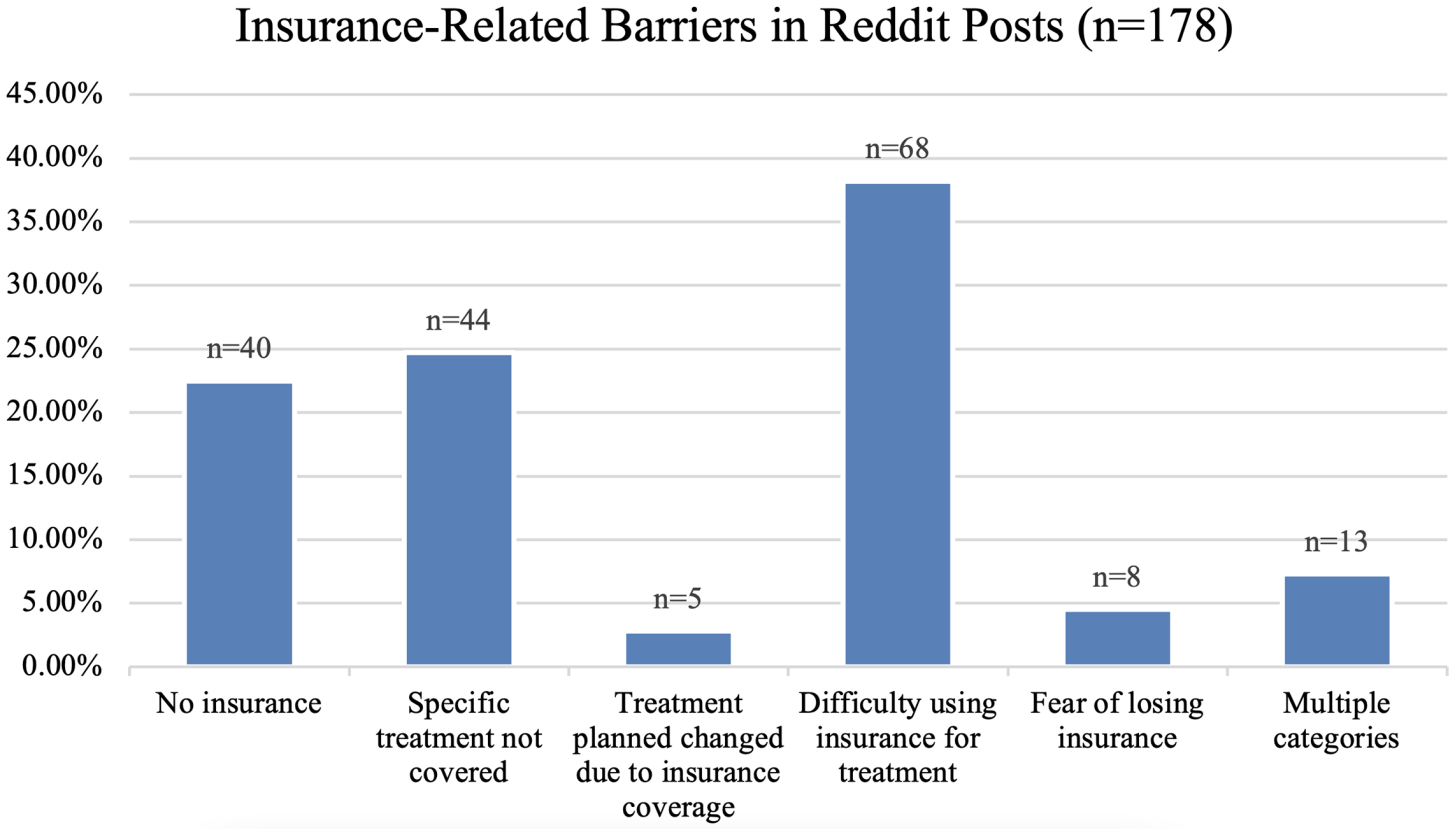
Breakdown of Insurance Related Barriers found in Reddit Posts.

The posts detailed personal experiences related to seeking medication treatment such as “*I really do not exactly want to broadcast to the world that I’m on methadone. Not because I’m embarrassed but stigma sucks*” and “*My insurance is always either denying my script completely or asking for prior auths every other month. Please help me*.” Figure 1 presents the types of insurance-related barriers from Reddit and their frequencies in our sample. 22.5% (40/178) of the posts mentioned not being insured at all (e.g., *“I do not have a q script because I do not have insurance*.”) and 24.7% (44/178) mention having insurance coverage but specific OUD treatments were not covered or only partially covered (e.g., *“The biggest issue with this medication is the cost. It’s $5,000 at least and insurance does not want to pay*.”). 4.5% (8/178) are related to fear of losing insurance coverage (e.g., *“I worry about this all the time. That my doctor will die, and I will not find another who will take my insurance, or they’ll gouge me for a $500 visit.*”). 2.8% (5/178) mention being forced to switch treatments due to insurance coverage barriers (e.g., *“The only reason I switched from suboxone to tex is bc I lost my health insurance*.”), and 38.2% (68/178), the most frequent subcategory, are related to general difficulties faced when using insurance to pay for OUD treatment (e.g., *“Getting insurance to pay for a preauthorization was a nightmare*.”). 7.3% (13/178) of the posts fit multiple categories (e.g., *“When only name brand strips were available, they would tell be [sic] it cannot be refilled because they do not have it in stock…people treat Suboxone users like full blown heroin addicts*.”).

**Figure 2:**
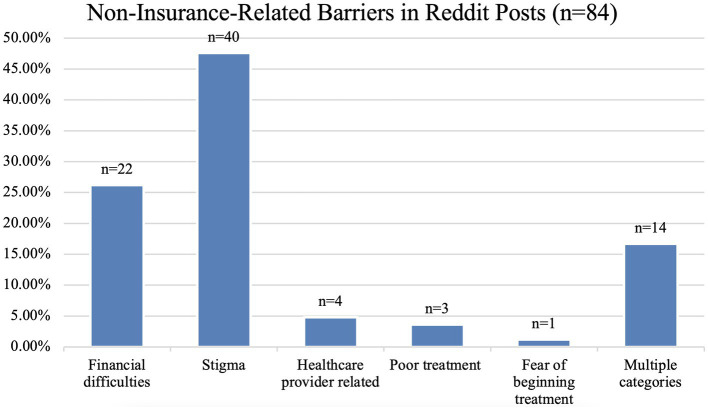
Breakdown of Non-Insurance Related Barriers found in Reddit Posts.

Figure 2 presents the breakdown of non-insurance barriers described by Reddit subscribers. 26.2% (22/84) of the posts are related to financial difficulties without any reference to insurance coverage (e.g., *“Sad that access is so expensive.”*), 47.6% (40/84) were related to stigma (e.g., *“I hate that we get the label as bad people or stupid for choosing to be an addict.”*), 4.8% (4/84) were attributed to issues with a healthcare provider such as difficulty finding care or regulations (e.g., *“What I’d really like to see is less requirements for getting take home medications to start with.”*), 3.6% (3/84) were related to poor treatment by either a clinician or pharmacist (e.g., *“I am shocked that I got snubbed like this*.”), and 1.2% (1/84) were related to the fear of beginning treatment (e.g., *“I was interested in a solution but I was too afraid to try it*.”). 16.7% (14/84) of the posts fit multiple categories (e.g., *“I have felt frustrated with many aspects of being on it such as urinalysis, snarky pharmacists, the cost, and often stigma associated with taking this*.”). The ‘financial difficulties’ category was only assigned to posts that specifically mentioned difficulties associated with finances without referring to insurance coverage. It is possible, and even likely, that the financial difficulties described by the subscribers were related to their insurance coverage in some way. Also, in our interpretation, descriptions of poor treatment by a clinician or pharmacist were considered to be external stigma.

### 3.2. Published literature

From the 362 papers identified in the literature search, we selected 19 publications for detailed review. Since the review was not conducted following the protocols for systematic reviews, it is likely that it does not comprehensively cover all barriers reported in the literature. However, we deemed this number to be sufficient for obtaining an estimate of the distribution of the reported barriers and their contents. As detailed in Table 1, stigma was the most noted barrier and was found in 78.9% (15/19) of the analyzed publications. Aside from stigma, the barriers mentioned were logistical issues (73.7%, 14/19), gender-specific barriers (36.8%, 7/19), fear (31.5%, 6/19), and lack of knowledge (26.3%, 5/19). 84.2% (16/19) of the included papers described the burdens of barriers on targeted population subsets or minorities. Out of the 16 publications that studied targeted populations, women represented 43.8% (7/16) and ethnic or racial minorities made up 31.3% (5/16). Recurring themes for the barriers affecting women were impacts on their children, lack of childcare, and the inability to focus on their recovery (42). Further details about the barriers reported are presented in Table 1.

**Table 1:** Summary of barriers identified from the literature, the associated papers, and their years of publication.

Publication title	Year	Sample size and description	Barriers
Treatment barriers identified by substance abusers assessed at a centralized intake unit (53)	2006	*N* = 312; individuals with substance use disorder at a centralized intake unit.	Belief that they do not have a serious issue
			Do not want spouse to know
			Fear
			Logistical and financial issues
			Stigma
			Privacy concerns
Stigma as a barrier to substance abuse and mental health treatment (54)	2011	*N* = 1,474; individuals over the age of 18 with unmet need for substance use treatment (from NSDUH).	Stigma
Barriers to integrated treatment of substance abuse and trauma among women (55)	2014	*N* = 20; one substance use treatment provider and its staff, including counsellors, technicians and parenting professionals.	Lack of available resources to address needs of individuals who have history of other abuses
			Underlying issues that facilitate substance abuse are not resolved which hinders overall treatment compliance and retention
			Stigma that women would not take responsibility for their addiction
			Automatically assumed that patients will find loopholes.
Need for women-centered treatment for substance use disorders: Results from focus group discussions (42)	2018	*N* = 10; women from a community treatment program for mothers in Kingston, Canada.	Lack of available treatment options
			Increased fear for women
			Stigma of “you are a mother and an addict”
			Concern regarding potentially losing children or lack of childcare at facilities may lead to a failure to accurately report substance abuse patterns
Understanding barriers to specialty substance abuse treatment among Latinos (56)	2018	*N* = 54; white, Black, and Latino individuals with recent history of substance use disorder.	Stigma of being seen as a “drug user”
			Fear of receiving subpar treatment
			Fear of treatment not working
			Financial or logistical issues
			Cultural barriers (providers do not truly understand immigration hardships and will not prescribe effective treatment)
			Treatment is not culturally accepted.
A systematic review of rural-specific barriers to medication treatment for opioid use disorder in the United States (57)	2019	*N* = 15; studies focusing on consumer-focused barriers to opioid use disorder treatment. Sample sizes in the articles ranged from 51 to 23,998 (including individuals with substance use disorder and practitioners).	Overall lack of access to treatment providers in rural areas
			Low income
			Stigma held by healthcare providers that rural drug users cannot be trusted and/or are unmotivated to get clean
Barriers and facilitators to the use of medications for opioid use disorder: A rapid review (58)	2020	*N* = 40; studies involving buprenorphine (5 also included naltrexone).	Stigma (also internalized stigma)
			Healthcare provider logistical/other issues
			Poor treatment experiences
			Knowledge gaps/misinformation
			Financial reasons
			Some programs require that you fail out of an abstinence-based treatment program before you can receive medication-based treatment
Challenges for women entering treatment for opioid use disorder (59)	2020	Narrative review focusing on treatment-related challenges faced by women with opioid use disorder.	Childcare issues impacting access to treatment
			Increased risk of negative outcomes compared to men
			Partner violence
			Women specific physiological challenges
			Stigma of being a mother and an addict
			Provider stigma and bias that women are not caring for their child properly
Stigma of opioid use disorder and its indirect effects on student pharmacists’ perceptions and attitudes (60)	2020	*N* = 244; student pharmacists.	Stigmatized policies and language
			Negative language such as “junkie,” “clean” vs. “dirty”
			Placing regulatory barriers to treatment
			Patient labeling of “difficult to treat” and “drug seeking”
			Some providers refuse to work with OUD patients
Overcoming barriers: individual experiences obtaining medication-assisted treatment for opioid use disorder (61)	2020	*N* = 20; individuals with opioid use disorder seeking treatment in rural New Mexico.	Stigma of “once an addict, always an addict”
			Logistical and financial issues
			Gender related barriers (women having kids/pregnancy
			Men experience gender disparity that drives them to commit low level drug crimes to expedite access to treatment
			Overall hassle to get into and maintain a program
			Knowledge limitations
Racial inequity in medication treatment for opioid use disorder: Exploring potential facilitators and barriers to use (62)	2021	*N* = 6,374; medicaid enrollees with opioid use disorder in Allegheny County, Pennsylvania.	Time spent in jail and at court appearances
			Interaction with housing services
			Racial inequity
Gender disparities in access and retention in outpatient methadone treatment for opioid use disorder in low-income urban communities (63)	2021	*N* = 11,169; clients at methadone treatment programs in Los Angeles, California.	Longer waitlist times for women Family responsibilities Lack of childcare and other family supports Inflexible job schedules
			Lack of transportation
			Implicit bias among health care providers
Treatment access for opioid use disorder in pregnancy among rural and American Indian communities (64)	2021	*N* = 17: clinics in three rural Utah counties serving pregnant women.	Lack of MOUD knowledge by both patients and clinical staff
			Lack of providers
			Lack of established referral network
			Lack of clinical training to care for pregnant individuals
Medications for opioid use disorder during incarceration (65)	2022	N/A	Stigma from carceral staff
			Internalized stigma
			Knowledge gaps/misinformation
			Lack of MOUD access in carceral system
Medication for opioid use disorder treatment continuity post-release from jail: A qualitative study with community-based treatment providers (66)	2022	*N* = 36: medical, supervisory and administrative staff at MOUD programs that serve jail-referred patients.	Lack of carceral staff understanding of MOUD
			Lack of referrals
			Lack of proximity between treatment facilities and jails
			SDOH, including houselessness, unemployment, and transportation
			Poor care coordination and communication
Women-reported barriers and facilitators of continued engagement with medications for opioid use disorder (67)	2022	*N* = 20; women in Western Massachusetts who had received MOUDs for 90 days.	Community-level and interpersonal stigma
			Gender specific stigma, such as being accused of sex work or child neglect
			Internalized stigma
			Mistrust of MOUD and providers
			Fear of MOUD and side effects
A qualitative meta-synthesis of pregnant women’s experiences of accessing and receiving treatment for opioid use disorder (68)	2022	*N* = 9: articles focusing on psychological motivators and barriers for pregnant women with opioid use disorder.	Anxiety about baby’s health
			Fear of authorities’ involvement
			Stigma from treatment providers
A qualitative analysis of barriers to opioid agonist treatment for racial/ethnic minoritized populations (69)	2022	*N* = 41; adult patients with opioid use disorder not currently receiving MOUDS in Boston, Massachusetts.	Negative opinions and distrust of MOUD
			Internalized stigma
			Anticipated stigma from community
Racial/ethnic residential segregation and the availability of opioid and substance use treatment facilities in US counties, 2009–2019 (70)	2022	Combined data from Substance Abuse and Mental Health Services Administration’s National Survey of Substance Abuse Treatment Services (2009, 2014, 2019) and 5-year American Community Surveys (2009, 2014, 2019).	More MOUD treatment facilities in counties with less interaction between Black and White residents (greater segregation)

## 4. Discussion

To the best of our knowledge, this is the first paper that attempts to enhance our existing understanding of the barriers to OUD treatment using two data sources, one of which is social media. Broadly speaking, we found many similarities in the barriers reported and some aspects in which the two sources can complement each other. In contrast to the literature, the Reddit posts did not frequently mention culture-specific barriers and only contained minor references to gender-specific barriers. We had intentionally targeted our literature search to cover specific subpopulations (gender and culture-specific), and hence, publications involving cultural and gender-specific barriers were well represented in our literature review. We were unable to devise comprehensive NLP methods that could identify these specific pieces of information from the Reddit posts, and thus, such information is somewhat under-represented in the posts we reviewed. The same is perhaps true for location- or transport-related barriers. While the literature revealed logistics, transport, and location (e.g., rural areas), these barriers were more difficult to detect from social media. It is unclear if such information is truly underrepresented in social media data or whether our NLP methods were unable to pick up such chatter. We did not perform further fine grained subpopulation or cultural analysis (43) in this study. It is also possible that the sphere of social media, including Reddit, itself represents a subculture and thus other cultural identities of the posters are of less importance.

The lack of specific MOUD treatment coverage despite access to health insurance was heavily discussed in both Reddit and the literature. Somewhat contrastingly, lack of insurance was a prominent theme on Reddit but not in the reviewed literature. While it is possible that our literature review was biased toward publications about non-insurance-related barriers due to its non-systematic nature, it is also possible that health insurance-related barriers are under-discussed in the literature. Clinical studies on the topic often include patients in non-emergency hospital settings and/or those with electronic health records, which may result in the exclusion of the population subset without insurance coverage who may not have access to healthcare. As illustrated by the examples presented in the *Results* section, many of the Redditors discussed gaps in insurance, reporting that their insurance would not cover treatments such as buprenorphine, or the cost would be too high despite having health insurance. It is also important to note that while we observed repetitions in the patterns of barriers that were mentioned in the literature, we did not formally evaluate the saturation of themes.

Stigma was a well-described barrier to care across both data sources and has been well-covered in past national initiatives as well (44). We now have a better understanding of how internalized stigma and awareness of stigma may be linked to negative psychosocial consequences (45) and may lead to secondary complications, such as depression and an elevated risk of overdose. When people with OUDs internalize or anticipate the public stigma attached to their illness, maladaptive behaviors leading to poorer health outcomes may occur (46), including general psychological distress and reduced desire to seek and maintain care often follow. This was found more so in individuals who had a prior history of seeking care that eventually failed and may have already experienced discrimination by both healthcare providers and others in their lives (47). The stigma-related discussions found on Reddit largely support what is known from the literature, and the richness of the information shared by the Redditors suggests that there is the potential of leveraging this resource to conduct in-depth analyses of stigma and its impact on the community in the future.

More research is particularly needed to understand the potential correlation between stigma held by healthcare providers and patient outcomes. In addition to administrative loopholes that must be overcome, healthcare providers also often perpetuate stigma toward those seeking medication treatment for OUD (48). Classifying OUD treatment as outside of “mainstream healthcare” coupled with a lack of awareness and uncertainty regarding treatment options often result in stigma which manifests as low empathy, low-quality care, or even denial of care (49). Structural stigma ultimately results in poorer outcomes by not only limiting access to care but by reinforcing internalized stigma felt by the patient. Historically, national campaigns to raise awareness have proven successful in reducing the stigma against individuals with HIV and mental health conditions and may also be successful for those with OUD. Other initiatives such as patient-centered programs, policy-focused efforts, and combating stigma head-on may also help to eliminate stigma (49, 50). Our study adds to the body of knowledge that exists about the importance and impact of stigma in the OUD-related epidemic and harm reduction efforts, and its findings highlight the need for substantial future research on the topic.

### 4.1. Utility and opportunity based on social media

The target population of this study (i.e., people with OUD), in addition to being hard to reach, is marginalized and discriminated against, which causes them to be at more risk of experiencing drug-related harm. To execute harm reduction efforts effectively, it is not only important to be aware of the barriers that exist for this population, but also to stay up to date with the evolving spectrum of barriers. Social media-based knowledge extraction methods, such as those leveraging NLP, have the potential to serve as a sustainable complementary mechanism for assessing and understanding barriers in the long run. It has the potential of including perspectives directly from people who use drugs, regardless of environmental, political, or geographical implications.

There is also the possibility of using social media as a platform for providing telehealth services. Since subscribers on social networks such as Reddit proactively seek help or support through these channels, there is the potential of taking healthcare services to them rather than expecting them to initiate treatment. Telehealth services may provide a comfortable environment for those seeking treatment without the burden of an in-person appointment (51). Social media-driven telehealth services may eliminate barriers including but not limited to travel, childcare, and subsequent money spent. Since flexibilities allowing telehealth for OUD treatment were put in place at the beginning of the COVID-19 pandemic, there has been increased treatment follow-up likely because treatment was received “on their own terms” (52). Social media-based telehealth services have enormous potential but this area of research remains underexplored. Future research should explore these opportunities and also the possibility of utilizing other social networks in addition to Reddit.

### 4.2. Limitations

While being convenient sources of information, social media data may potentially be non-representative of the US population (e.g., overrepresentation of younger people), and may contain posts generated by bots as opposed to real people. The latter, however, was not the case in the sample we reviewed. Subreddits on Reddit are strictly moderated by volunteers, which leads to the prompt removal of posts that violate community rules. Another important limitation is that samples from social media only describe barriers that are perceived by patients and may not capture additional barriers that patients would not necessarily realize are impacting their care (e.g., structural, social, or self-stigma/barriers). Finally, the distributions of barriers are unlikely to be identical for the three medications included in this study. We did not attempt to group the distributions by substance or perform in-depth analyses of the impacts of multiple barriers. There are also limitations associated with our literature review. The review focused on selecting a small sample of relevant papers, and the protocols for conducting systematic reviews were not followed. Although the selected papers were filtered to fit our study based on relevance, the limitation of a small selection is present. By expanding both the subject matter of the Reddit queries to include topics such as structural barriers and increasing the number of papers for review, we can hope to extend this work to cover broader perspectives of barriers to treatment. Our review also did not cover any strategies implemented around the world to tackle these barriers, and we leave such a review as future work.

## 5. Conclusion

In this paper, we described barriers to OUD treatments reported on Reddit and compared them to those reported in published literature. Similar barriers were reported in both sources and included stigma, lack of insurance, lack of adequate coverage for OUD treatment options, financial challenges, and personal fears about accessing healthcare. Our study shows that social media platforms such as Reddit serve as important communication channels for people with OUD to discuss treatment-related barriers. In the continuously evolving sphere of substance use and substance use disorder, harm reduction strategies can leverage information from social media-based sources to identify sources of barriers and mitigate their impacts.

## Data availability statement

The original contributions presented in the study are included in the article/supplementary material, further inquiries can be directed to the corresponding author.

## Author contributions

WB conducted qualitative analysis of the data, conducted part of the literature review, and prepared the manuscript draft. KP conducted part of the literature review, quantified the statistics provided in the paper, and aided the preparation of the draft. DW, MB, and JL aided the qualitative analyses, contributed to the preparation of the manuscript, and provided domain expertise. JP conceived the study ideas and provided domain expertise. AS conducted the technical aspects of the manuscript. All authors contributed to the preparation of the final manuscript and approved the final version.

## Funding

Research reported in this publication is supported by the National Institute on Drug Abuse (NIDA) of the National Institutes of Health (NIH) under the award number R01DA057599. The content is solely the responsibility of the authors and does not necessarily represent the official views of the NIH.

## Conflict of interest

The authors declare that the research was conducted in the absence of any commercial or financial relationships that could be construed as a potential conflict of interest.

## Publisher’s note

All claims expressed in this article are solely those of the authors and do not necessarily represent those of their affiliated organizations, or those of the publisher, the editors and the reviewers. Any product that may be evaluated in this article, or claim that may be made by its manufacturer, is not guaranteed or endorsed by the publisher.
